# Supplementary material for: Prognostic Value of the Neutrophil-to-Lymphocyte Ratio before and after Radiotherapy for Anaplastic Thyroid Carcinoma
Source: Cancers (Basel). 2021 Apr 15;13(8):1913. doi: 10.3390/cancers13081913 (PMC8071431; doi:10.3390/cancers13081913)
Supplement: Supplementary file 1 [file cancers-13-01913-s001.zip › cancers-1151525-supplementary.pdf]

Supplementary Materials: Prognostic Value of the Neutrophil-to-Lymphocyte Ratio before and After Radiotherapy for Anaplastic Thyroid Carcinoma

Jiyun Park, Jun Park, Jung Hee Shin, Young Lyun Oh, Hyun Ae Jung, Man Ki Chung, Jun-Ho Choe, Yong Chan Ahn, Sun Wook Kim, Jae Hoon Chung, Tae Hyuk Kim and Jae Myoung Noh

Table S1. Overall survival according to the total radiation dose (based on 60 Gy) in patients with available complete blood cell count data after radiotherapy for anaplastic thyroid carcinoma.

Survival	EQD2$_{10}$	
	**<60 Gy**	**≥60 Gy**
Died	12	5
Alive	1	7

EQD2$_{10}$, equivalent dose in 2 Gy fractions with an α/β ratio of 10.

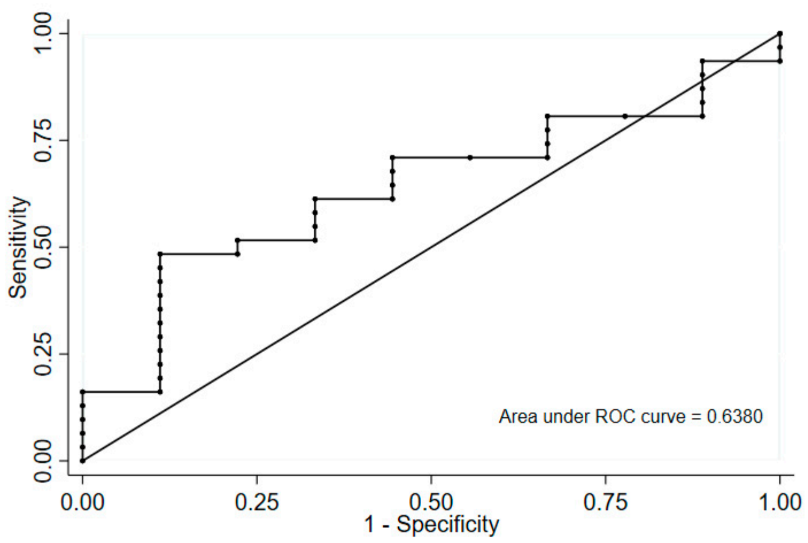

Figure S1. ROC curve assessing the cutoff points of baseline NLR. ROC, receiver operating characteristics; NLR, neutrophil-to-lymphocyte ratio.

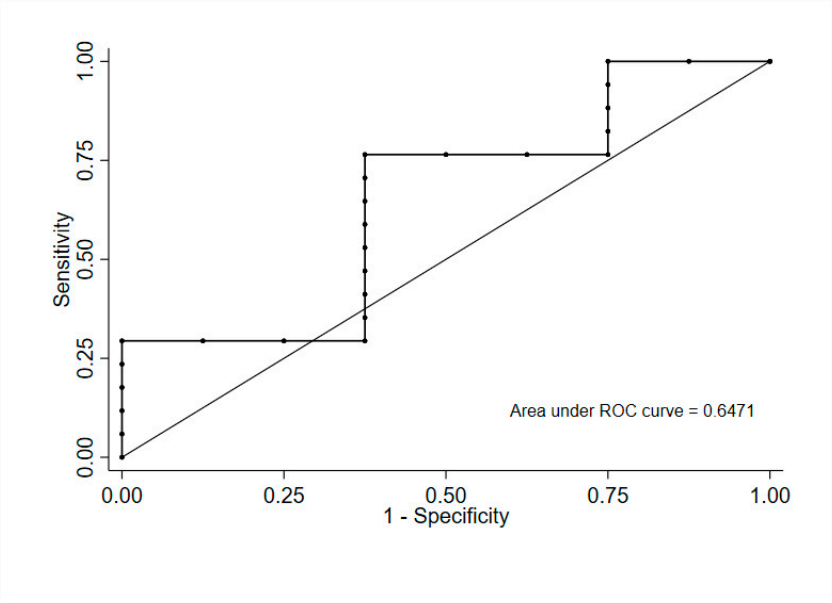

Figure S2. ROC curve assessing the cutoff points of post-RT-NLR. ROC, receiver operating characteristics; RT, radiation therapy; NLR, neutrophil-to-lymphocyte ratio.
